# CRISPR/Cas9 gene editing: A new hope for transthyretin amyloidosis treatment

**DOI:** 10.1016/j.amsu.2022.104784

**Published:** 2022-09-28

**Authors:** Faizan Shahzad, Mohammad Ebad Ur Rehman, Jawad Basit, Sajeel Saeed, Khawar Abbas, Muhammad Farhan

**Affiliations:** Department of Medicine, Rawalpindi Medical University, Block E Satellite Town, Rawalpindi, Pakistan; Department of Surgery, Rawalpindi Medical University, Block E Satellite Town, Rawalpindi, Pakistan; Department of Surgery, Rawalpindi Medical University, Block E Satellite Town, Rawalpindi, Pakistan; Department of Medicine, Rawalpindi Medical University, Block E Satellite Town, Rawalpindi, Pakistan

**Keywords:** Gene editing, CRISPR, Amyloidosis

To The Editor

Transthyretin amyloidosis (ATTR Amyloidosis) is a disorder in which a misfolded protein called transthyretin (TTR) gradually accumulates [1]. It can lead to both neuropathy and cardiomyopathy (ATTR-CM) [2]. ATTR-CM leads to heart failure, with patients reporting fatigue, shortness of breath, and edema [3]. Neuropathic (NP) hereditary transthyretin amyloidosis (hATTR) was reported for the first time in 1952 [4]. Amyloid deposits can be found in the peripheral nerves, cardiovascular system, and kidneys [5]. Amyloid fibrils cause direct damage to Schwann cells [6]. The classical presentation shows an earlier onset of neuropathy and prominent small fiber involvement. Patients can also develop distal sensory loss. Later on, larger myelinated nerve fibers are affected which can hinder the modalities of light touch, vibration, and progressive motor skills [7]. The diagnosis should be confirmed by checking for monoclonal protein [8]. DPD, PYP, and HMDP are recommended biotracers for ATTR diagnosis [9]. The available treatment options for ATTR, such as diflunisal or tafamadis, stabilize the TTR protein in its tetrameric form, leading to a decrease in the formation of amyloid [10]. However, all therapies require long-term administration, which may exacerbate adverse effects [11].

Clustered regularly interspaced palindromic repeats (CRISPR) or Cas9 is a gene-editing technology that has promised to revolutionize biomedical research [12] CRISPR may be utilized for gene therapy, curing infections and the treatment of cancer. CRISPR/Cas9 technology consists of Cas9 endonuclease and a guide RNA that allows the genome to be altered by causing a double-stranded DNA break [13]. The current treatments are not as effective as there is a need to administer them for long periods to maintain sufficient TTR knockdown. ATTR amyloidosis is an ideal candidate for the application of CRISPR, as a single gene mediates disease pathogenesis. Knockdown of the gene coding the TTR protein minimally affects the normal physiological processes [14]. NTLA-2001 is a new intravenous CRISPR-Cas9–based therapy. It is administered by an intravenous infusion. It can reduce both wild-type and mutant TTR after a single dose by editing TTR in hepatocytes [15]. The NTLA-2001 consists of a lipid nanoparticle (LNP) delivery system with an affinity for liver tissue. It contains two components; the first is a single guide RNA that targets TTR and the second is an mRNA sequence of *Streptococcus pyogenes* Cas9 protein [16]. By keeping in mind the lipid composition and an efficient way of delivery to the liver via the RNA cargo, the LNP formulation for this new CRISPR therapy was developed. Plasma apolipoprotein-E binds to the LNP surface in blood. Hepatocytes endocytose the LNP via LDL receptor [17]. As NTLA-2001 is specific to the liver, it has minimal systemic adverse effects while maintaining excellent efficacy, since the liver is the primary site of TTR formation [18].

A clinical trial on mice resulted in greater than 97% knockdown of the mouse TTR. A biodegradable lipid was utilized to create LNP-INT01, instead of viral delivery systems. Viral systems are not cleared from the body rapidly and lead to sustained action. However, this biodegradable system was readily cleared from the body and by the third day of administration, it had reached undetectable levels. The Cas9 protein levels reached a maximum level approximately 4 hours after infusion. LNP-INT01 was injected into the mice at doses ranging from 0.3 to 3 mg/kg. The follow-up period was 1 year and a sustained decrease in TTR levels was reported. LNP-INT01 is currently the most efficient delivery method for CRISPR [19]. A newer study demonstrated reasonable effectiveness of non-viral administration of Cas9 mRNA along with viral delivery of the guide RNA. However, knockdown was approximately 25% and significant immune response was a concern [20]. In a study, 6 human participants were enrolled. On day 28, a TTR reduction of 52% in the group that received 0.1 mg/kg was noted, and an 87% reduction in the group that received the lowest dose. The adverse effects were minimal and those that did occur, were mild. They were reported in 3 (50%) participants. In 5 (83%) patients, increased D-dimer levels were seen 24 hours after administration. The values returned to normal after 7 days. Liver function measures remained within normal levels and coagulation measures also remained reasonably well [15].

CRISPR therapy promises to revolutionize the treatment of several hematological diseases, with ongoing trials focusing on leukemia, lymphoma, and thalassemia. ATTR amyloidosis is a life-threatening disease of the heart or nerves; all available therapies require long-term administration. Further research into the role of CRISPR in ATTR therapy is essential in making progress towards the establishment of a permanent cure for this disease.

## Ethical approval

Not applicable.

## Please state any sources of funding for your research

None.

## Author contribution

Faizan Shahzad: Study conception, write-up, critical review and approval of the final version, Mohammad Ebad Ur Rehman: Study conception, write-up, critical review and approval of the final version, Jawad Basit: Study conception, write-up and approval of the final version, Sajeel Saeed: Study conception, critical review and approval of the final version, Khawar Abbas: Study conception, critical review and approval of the final version, Muhammad Farhan: Study conception, critical review and approval of the final version.

## Consent

Not applicable.

## Registration of research studies

1. Name of the registry: Not applicable
2. Unique Identifying number or registration ID: Not applicable
3. Hyperlink to your specific registration (must be publicly accessible and will be checked): Not applicable

## Guarantor

Mohammad Ebad Ur Rehman.

## Declaration of competing interest

The authors have no conflicts of interest.
